# Vitamin D Receptor *TaqI* Polymorphism Is Associated With Reduced Follicle Number in Women Utilizing Assisted Reproductive Technologies

**DOI:** 10.3389/fendo.2018.00252

**Published:** 2018-05-28

**Authors:** Mila W. Reginatto, Bartira M. Pizarro, Roberto A. Antunes, Ana C. A. Mancebo, Luísa Hoffmann, Pâmela Fernandes, Patrícia Areas, Maria I. Chiamolera, Rosane Silva, Maria do Carmo Borges de Souza, Enrrico Bloise, Tânia M. Ortiga-Carvalho

**Affiliations:** ^1^Instituto de Biofisica Carlos Chagas Filho, Universidade Federal do Rio de Janeiro, Rio de Janeiro, Brazil; ^2^Fertipraxis – Centro de Reprodução Humana, Rio de Janeiro, Brazil; ^3^Maternidade Escola, Universidade Federal do Rio de Janeiro, Rio de Janeiro, Brazil; ^4^Departmento de Medicina, Escola Paulista de Medicina, Universidade Federal de São Paulo, São Paulo, Brazil; ^5^Departamento de Morfologia, Universidade Federal de Minas Gerais, Minas Gerais, Brazil

**Keywords:** calcitriol, ***VDR*** polymorphisms, 25(OH)D, *TaqI*, folliculogenesis, infertility

## Abstract

**Purpose:**

Calcitriol, or 1,25-hydroxycholecalciferol, is the active form of vitamin D. It binds and activates vitamin D receptor (VDR). Infertility and defective folliculogenesis have been observed in female *vdr-*knockout mice; however, whether *VDR* polymorphisms affect human ovarian responses to controlled ovarian stimulation (COS) remains unclear. We hypothesized that *VDR* polymorphisms are associated with infertility and COS responses. Thus, we evaluated the association between the *TaqI, BsmI*, and *FokI VDR* polymorphisms and ovarian responses in women undergoing COS.

**Methods:**

In this study, we recruited a control group (*n* = 121) comprising volunteers with a history of natural conception and a second group of women undergoing COS (*n* = 70). *TaqI, BsmI*, and *FokI* genotyping was performed *via* restriction fragment length polymorphism analysis or TaqMan qPCR and Sanger sequencing. Intrafollicular 25(OH)D contents were measured in follicular fluid collected from COS patients during oocyte retrieval. Ovarian response parameters were obtained from patient medical records.

**Results:**

There were no significant differences in the genotype frequencies of *VDR* polymorphisms (*TaqI, BsmI* and *FokI)* between the control and COS groups. However, the allele frequency of *TaqI* (C allele) was significantly lower in the COS group than in the control group (*p* = 0.02). Follicle number but not oocyte number was lower in patients with *TaqI* polymorphic (TC/CC) genotypes (*p* = 0.03). Importantly, the ratio between the number of follicles retrieved and intrafollicular estradiol concentrations was higher in patients with the TC/CC *TaqI* genotypes (*p* < 0.02).

**Conclusion:**

We identified an association between the *VDR TaqI* polymorphism and reduced follicle number in women undergoing COS, suggesting that *VDR* signaling affects the ovarian response to stimulation *via* unknown mechanisms.

## Introduction

Calcitriol, or 1,25-hydroxycholecalciferol (1,25(OH)2D), is the active form of vitamin D, a steroid hormone that exerts classical functions in calcium and phosphorus homeostasis and bone mineralization (1). Calcitriol binds its nuclear receptor, vitamin D receptor (VDR) (2), and has an array of actions in the immunological, cardiovascular (3), and reproductive systems of both genders (4). In particular, a number of studies have demonstrated an association between 25-hydroxyvitamin D (25(OH)D) concentrations and different causes of infertility in animals (3, 5–8) and humans (9–14).

*VDR* expression has been reported in different central (hypothalamus and hypophysis) and peripheral reproductive organs (ovary, uterus, placenta, and oviduct) (13, 15, 16). Evidence linking calcitriol and reproductive function has been demonstrated in 7-week-old female *vdr-*knockout mice. These animals exhibited uterine hypoplasia, defective folliculogenesis (the absence of mature follicles), and associated infertility (6, 7). Moreover, female *vdr-*knockout mice exhibited decreased aromatase expression and activity in the ovary, and these effects were associated with elevated serum luteinizing hormone (LH) and follicle-stimulating hormone (FSH) concentrations, indicating a peripheral rather than a central defect (8) and suggesting a role for calcitriol in regulating folliculogenesis.

Genetic alterations in the *VDR* gene may lead to important defects in gene activation. Alterations were reported to affect calcium metabolism, cell proliferation, and immune function (17). Furthermore, some *VDR* single-nucleotide polymorphisms (SNPs) may contribute to a genetic predisposition to certain diseases. SNPs present in the *VDR* gene alter receptor length and decrease its activation in target cells (18). Among these polymorphisms, the best described are *TaqI, BsmI*, and *FokI*. *TaqI* (rs731236, changes T/C, exon 9) and *BsmI* (rs1544410, changes G/A, intron 8) are present in the 3′ untranslated region (3′ UTR) of the *VDR* gene and are related to modulation of gene and protein expression of the receptor. The *FokI* polymorphism (rs2228570, changes T/C, exon 9), in turn, is present in a translated region and effects functional activity by generating a longer VDR protein with reduced transcriptional activity (19).

These polymorphisms have been previously associated with increased risk of developing diabetes (20), tuberculosis (*FokI* polymorphism) (21), specific cancers (22, 23), and multiple sclerosis (24). Conversely, they were also associated with protection against breast cancer (*TaqI* polymorphism) (25), osteoporosis (26), and asthma (*FokI* polymorphism) (27). However, no associations between *VDR* polymorphisms have been reported in conditions such as osteoporosis (28), colorectal cancer (29), and metabolic syndrome (30). In the context of reproductive medicine, *VDR* polymorphisms have been associated with polycystic ovarian syndrome and endometriosis (17, 31–38), although these results are inconclusive and require further investigation.

25(OH)D deficiency is now recognized as a pandemic condition (39). In Brazil, 25(OH)D deficiency is largely detected in women of different ages, including elderly and postmenopausal women (40) and women of reproductive age (41). Controlled ovarian stimulation (COS), which aims to increase the success rate of *in vitro* fertilization (IVF) through stimulation of folliculogenesis, revealed a decrease in the pregnancy (42) and fertilization rates (43) in women with lower 25(OH)D concentrations.

Moreover, other studies have demonstrated that women with replete serum concentrations of 25(OH)D (42) or at least sufficient 25(OH)D in the follicular fluid (FF) had lower pregnancy and fertilization rates (44). A recent study from our group demonstrated that women with lower follicular 25(OH)D concentrations exhibited better outcomes when treated with the COS protocol in that they produced more larger follicles and had higher serum estradiol concentrations (45). Despite these controversial data, *in vitro* and animal model studies strongly support a significant role of calcitriol in orchestrating reproductive processes and IVF outcomes (46). However, further studies are warranted to demonstrate a causal relationship between 25(OH)D status and infertility.

In the present study, we hypothesized that *VDR* polymorphisms are associated with infertility and response to COS. The identification of specific *VDR* polymorphisms that can be shown to be related to infertility and response to COS may help clarify the causes underlying female infertility and poor ovarian response.

## Materials and Methods

### Patients

Two groups of patients were enrolled for each polymorphism analysis. The control group comprised volunteer women with no history of reproductive disorders. To be included in the control group, volunteers had to declare that they had become pregnant through natural conception at least once and had never experienced any difficulties in conceiving. The COS group consisted of women who underwent COS for intracytoplasmic sperm injection (ICSI) at the Fertipraxis Center for Human Reproduction, a clinic certified by the Brazilian Health Surveillance Authority (ANVISA) and the Latin American Network of Assisted Reproduction (REDLARA). We enrolled 62 controls and 47 COS-treated women in the *TaqI* polymorphism analysis, 57 controls and 49 COS-treated women in the *FokI* analysis, and 86 controls and 54 patients in the *BsmI* analysis.

This study was approved by the local Ethics Committee and was registered on the Brazilian platform of research under the number 02213812.4.0000.5275. All the enrolled subjects (volunteers and patients) provided written informed consent before joining the study. In the COS group, clinical data, including hormone concentrations [serum and follicular estrogen (E2), progesterone (P4), LH, and FSH] and indicators of ovarian response (number of follicles and oocytes retrieved), were obtained from patient medical records. Clinical data for the control group were obtained during patient enrollment and interviews. All patients underwent blood collection for further *VDR* polymorphism genotyping.

### COS Protocol

Controlled ovarian stimulation protocols were performed according to the specific clinical requirements of the patients. Briefly, the gonadotropin-releasing hormone antagonist analog cetrorelix acetate (Cetrotide^®^ 0.25 mg, Merck-Serono, Italy) was administered to induce hypophysis suppression, and on the second day of menstruation, ovarian stimulation was initiated with synthetic FSH alone (Gonal-F^®^, Merck-Serono, Italy; or Bravelle^®^, Ferring Pharmaceutical, Germany) or FSH and LH (Pergoveris^®^, Merck-Serono, Italy; or Menopur^®^, Ferring Pharmaceutical, Germany) treatments. FSH dosage varied from 150 to 300 IU/day, and LH dosage ranged from 75 to 300 IU/day.

When at least one follicle had reached 18 mm or at least two follicles had reached 16 mm (assessed by ultrasound), human chorionic gonadotropin (hCG) (Ovidrel^®^ 250 μg, Merck-Serono, Italy) was administered to mimic LH. Thirty-five hours post-Ovidrel^®^ administration, the oocytes were retrieved, and FF was obtained during the follicular aspiration procedure. In addition, blood samples were collected for *VDR* genotyping following FF isolation.

### DNA Extraction

Blood (1 ml) was submitted for genomic DNA extraction from peripheral leukocytes *via* the salting-out technique (47) using a commercial Wizard^®^ Genomic DNA purification kit according to the manufacturer’s instructions (A1120, Promega, Madison, WI, USA). After the extraction, DNA quantity and quality were examined using a NanoPhotometer (Implen, Munchen, Germany).

### Genotyping

The genotyping of *TaqI* (rs731236) and *FokI* (rs2228570) polymorphisms was performed using the restriction fragment length polymorphism (RFLP) technique. Table 1 shows the primer sequences used for the *VDR* polymorphism analysis, which were validated through the Primer Blast program to ensure PCR quality; intron-spanning primers were used to avoid contamination with external genomic DNA. To perform the PCR reactions, a commercial kit (GoTaq, Promega, USA) was used and the conditions were as follows: 95°C for 4 min, 35 cycles of 95°C for 30 s, 60°C for 30 s, 72°C for 1 min, and 72°C for 7 min. DNA samples were digested by *TaqI* and *BseGI* (*Btscl* isoschizomers that recognize the same sequence recognized by the *FokI*) endonucleases (Thermo Scientific, EUA). The mixtures were incubated at 65°C and 55°C, respectively, to promote cleavage. The samples were then subjected to electrophoresis on 2–4% agarose gels to determine the lengths of the fragments and genotyping results (Figures S1 and S2 in Supplementary Material).

**Table 1:** Sequences of primers used to amplify each polymorphism and their respective fragments with or without endonucleases.

Polymorphism	Sense	Antisense	Length	Reference
*TaqI* (rs731236)	CAGAGCATGGACAGGGAGCAA	GCAACTCCTCATGGCTGAGGTCTC	495 bp uncut	(48)
			290, 245 bp cleaved	
*FokI* (rs2228570)	AGCTGGCCCTGGCACTGACTCTGCTCT	ATGGAAACACCTTGCTTCTTCTCCCTC	265 bp uncut	
			196, 69 bp cleaved	

*Pb, pair of bases*.

*BsmI* (rs1544410) polymorphism genotyping was performed using TaqMan Genotyping Master Mix (Applied Biosystems, Foster City, CA, USA, 4371355) and a TaqMan^®^ SNP Genotyping Assay (Applied Biosystems, PN4351379) in a ViiA™ 7 Real-Time PCR System (Applied Biosystems, Foster City, CA, USA). Allele discrimination was analyzed using the ViiA™ 7 software, and genotyping was performed with Genotyping version 3.1 from Thermo Fisher Cloud. Furthermore, Sanger sequencing (Big Dye^®^ Terminator v 3.1 Cycle Sequencing Kit) was performed on the four amplified products for which real-time PCR did not achieve accurate results to confirm the genotyping assay results (Figure S3 in Supplementary Material). The same primers used for amplification were used for genotyping assessment (5′CAACCAAGACTACAAGTACCGCGTCAGTG3′ and 3′AACCAGCGGGAAGAGGTCAAGGG5′) with 1 cycle at 96°C for 1 min, 25 cycles at 96°C for 15 s, 50°C for 15 s, and 60°C for 4 min. Products of the sequencing reactions were assessed in a Genetic Analyzer ABI3500. Sequence analysis was performed using MacVector, version 14.

### FF Collection for 25(OH)D Measurement

Follicular fluid collection was performed during oocyte capture, as previously described (45, 49). Briefly, follicle aspiration was undertaken with a transvaginal ultrasound probe as a guide (Medison X8^®^) and a 17G oocyte aspiration needle (Wallace^®^) connected to a closed vacuum system under 90 mmHg of negative pressure, which was used to empty the follicles. The follicle exhibiting the largest diameter, greater than 16 mm, was selected, captured and placed in a sterile container. FF was extracted after oocyte detection and subsequently frozen in liquid nitrogen. This technique allowed the collection of fluid from a single follicle and decreased the chance of blood contamination.

### Quantification of 25(OH)D

FF 25(OH)D concentrations were assessed using an electrochemiluminescence fixation assay (ElecsysTotal Vitamin D total assay, Roche Diagnostics, Brazil). The range of measurements was 3–70 ng/ml. Inter- and intra-assay variations were 5.9 and 5.2%, respectively. This technique is based on competition, and a vitamin D-binding protein binds 25-hydroxycholecalciferol (25(OH)D3) and 25-hyroxyergocalciferol (25(OH)D2).

### Statistical Analysis

Genotype and allele frequencies were calculated based on the observed genotypes. Departure from Hardy–Weinberg equilibrium (HWE) in the distribution of the genotypes was estimated with the χ^2^ test. If the χ^2^ test resulted in a *p* value greater than 0.05, the population was considered to be in HWE. The influence of each *VDR* polymorphism on COS variation was assessed by an odds ratio (OR) analysis. We performed χ^2^ tests to analyze heterogeneity, and a value of *p* < 0.05 was considered to indicate statistical significance. The dominant model, in which heterozygous and homozygous minor alleles were grouped, was analyzed.

The Mann–Whitney test was used to investigate possible associations between polymorphisms and ovarian response variables and to test associations between polymorphisms and FF concentrations of 25(OH)D. A *p* value <0.05 was considered statistically significant. All comparisons were performed using SPSS (version 22) software. Graphics were generated using Prism (version 6) software.

## Results

### Clinical Data

To determine whether the presence of polymorphisms in the *VDR* gene affected the ovarian response of women undergoing COS, we extracted clinical data from control volunteers who had declared a history of natural conception and from women who underwent the COS protocol and ICSI treatment. The demographic parameters of the control and COS groups are depicted in Table 2.

**Table 2:** Demographic parameters of control and COS groups.

	Control	COS	*p-*Value
Age (years)	44 ± 0.9	35 ± 0.5	<0.0001
Height (cm)	161 ± 0.7	164 ± 0.7	<0.01
Weight (kg)	66 ± 1.0	61 ± 1.2	<0.002
BMI (kg/m^2^)	25.5 ± 4.1	22.5 ± 3.9	<0.0001

*BMI, body mass index; COS, controlled ovarian stimulation*.

The COS infertility diagnoses in our group were as follows: unexplained (37%), tubal factors (18%), ovarian failure (13%), endometriosis (13%), female anatomical causes (6%), and other causes of infertility (13%), including hypogonadism, colonic surgery, ovarian failure and tubal factors, female endocrine factors, breast cancer, tubal factors and endometriosis, or polycystic ovarian syndrome and endometriosis.

### Analysis of *TaqI, BsmI*, and *FokI VDR* Polymorphisms

Table 3 shows the genotype frequencies of the *VDR* polymorphisms studied in the control versus COS groups. No differences were found, and the *FokI* polymorphism was in HWE (control: *p* = 0.6, COS: *p* = 0.23). Table 4 shows the allele frequencies of the *VDR* polymorphisms studied in the control versus COS groups. No differences were observed in the *BsmI* and *FokI* allele frequencies between the control and COS groups. However, the *TaqI* polymorphism exhibited a higher frequency of the C allele and a lower frequency of the T allele in the COS group [*p* = 0.02; OR: 1.95 (1.097–3.5)]. We then applied the dominant model and identified a considerable trend in the genotype distribution for the *TaqI* polymorphism [*p* = 0.056, OR: 2.106 (0.979–4.53)].

**Table 3:** Genotype frequencies of *VDR* polymorphisms.

Polymorphism	Genotype frequencies (%)		χ^2^ (*p*)
	Control (*n*)	COS (*n*)	
***TaqI* (rs731236)**	*n* = 62	*n* = 47	
TT	62.9 (39)	42.6 (20)	4.47 (0.10)
TC	22.6 (14)	34.0 (16)	
CC	14.5 (9)	23.4 (11)	
*p* (HWE)	<0.01	0.13	
***FokI* (rs2228570)**	*n* = 57	*n* = 49	
TT	50.9 (29)	47.0 (23)	0.76 (0.68)
TC	36.8 (21)	35.0 (17)	
CC	12.3 (7)	18.0 (9)	
*p* (HWE)	0.60	0.23	
***BsmI* (rs1544410)**	*n* = 86	*n* = 54	
GG	54.7 (47)	42.6 (23)	2.22 (0.33)
GA	9.30 (8)	14.8 (8)	
AA	36.0 (31)	42.6 (23)	
*p* (HWE)	<0.01	<0.01	

*COS, controlled ovarian stimulation; HWE, Hardy–Weinberg equilibrium (*p* value < 0.05 is not considered in HWE; *p* value > 0.05 is considered in HWE). χ^2^—chi-squared test (values above 3.84; *p* < 0.05)*.

**Table 4:** Allele frequencies of *VDR* polymorphisms.

Polymorphism	Allele frequencies (%)		OR (95% CI)	χ^2^ (*p*)
	Control (*n*)	COS (*n*)		
***TaqI* (rs731236)**	*n* = 124	*n* = 94		
T	74.0 (92)	60.0 (56)	1.95 (1.097–3.5)	5.24 (0.02)*
C	26.0 (32)	40.0 (38)		
***FokI* (rs2228570)**	*n* = 114	*n* = 98		
T	69.0 (79)	64.0 (63)	1.25 (0.71–2.21)	0.60 (0.44)
C	31.0 (35)	36.0 (35)		
***BsmI* (rs1544410)**	*n* = 172	*n* = 108		
G	59.3 (102)	50.0 (54)	1.45 (0.90–2.35)	2.32 (0.12)
A	40.7 (70)	50.0 (54)		

*COS, controlled ovarian stimulation. OR, odds ratio. IC, confidence interval. χ^2^, chi-squared test (values above 3.84; *p* < 0.05)*.

*Significant p value.

### *TaqI* Polymorphism and COS-Related Variables/25(OH)D Associations

Because an association was detected between infertility and the frequency of *TaqI* alleles, we next examined whether the *TaqI* polymorphism is associated with variables related to the COS protocol. We, therefore, sorted the COS group according to genotype based on the dominant model (Table 5). A comparison of the *TaqI* genotypes did not reveal any differences in LH, FSH, E2, or P4 concentrations on day 1 of the COS protocol. However, there was a trend for women possessing the TC/CC genotypes to have a lower number of antral follicles than were found in women with the TT genotype (*p* = 0.08). The duration of COS and the FSH dose administered did not differ according to the *TaqI* genotype (Table 5). Similarly, a comparison of *TaqI* genotypes at baseline (Table 6) before COS revealed that there were no differences in 25(OH)D, E2, and P4 concentrations on the day of oocyte retrieval.

**Table 5:** Ovarian stimulation-related variables according to *TaqI* genotype.

		TT	TC/CC	*p*
Day 1 of COS	Antral follicles	13.1 ± 1.03	10.9 ± 0.74	0.08
	FSH (mU/ml)	6.5 ± 0.74	5.9 ± 0.56	0.54
	LH (mU/ml)	4.9 ± 0.59	6.5 ± 0.59	0.08
	E2 (pg/ml)	53.5 ± 7.2	83.1 ± 10.8	0.11
	P4 (pg/ml)	361 ± 43	340 ± 32.4	0.69
COS duration (days)		9.5 ± 0.45	10.0 ± 0.34	0.39
FSH (UI)		1835 ± 152	1941 ± 168	0.64

*COS, controlled ovarian stimulation; E2, estradiol; P4, progesterone; FSH, Follicle-stimulating hormone; LH, Luteinizing hormone*.

*The data are expressed as the medians ± SEM*.

**Table 6:** Intrafollicular 25(OH)D, E2 and P4 concentrations according to *TaqI* genotype.

		TT	TT/CC	*p*
Intrafollicular (capitation day)	25(OH)D	22.5 ± 3.1	25.6 ± 2.4	0.4
	E2 (ng/ml)	423 ± 91	488 ± 75	0.58
	P4 (pg/ml)	18.34 ± 2.7	23.87 ± 41.3	0.69

*E2, estradiol; P4, progesterone; 25(OH)D, 25-hydroxycholecalciferol*.

*The data are expressed as the medians ± SEM*.

**Figure 1:**
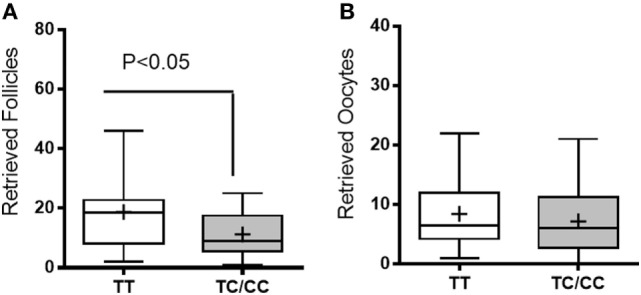
Follicles **(A)** and retrieved oocytes **(B)**. White bars represent women carrying the TT genotype, whereas gray bars represent women carrying the TC/CC genotypes. Unpaired *t*-test. The data are presented as the mean ± min and max.

**Figure 2:**
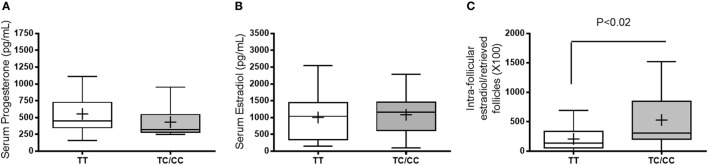
Serum concentrations of progesterone **(A)** and estradiol **(B)** on the day of human chorionic gonadotropin administration and the ratio of serum estradiol/to retrieved follicles **(C)**. White bars represent women carrying the TT genotype, whereas the gray bars represent women carrying the TC/CC genotypes. Unpaired *t*-test. The data are presented as the mean ± min and max.

We further analyzed the number of follicles and retrieved oocytes according to the *TaqI* genotypes (Figure 1). A lower number of mature follicles was found in women with the TC/CC genotypes than in women possessing the TT genotype (*p* = 0.03). However, we found no significant differences in the number of oocytes retrieved. We also analyzed the serum concentrations of E2 and P4 on the day of hCG administration as well as the ratio of intrafollicular E2 to follicles retrieved according to *TaqI* genotype (Figure 2). There were no significant associations between *TaqI* genotypes and serum concentrations of E2 or P4 on the day of hCG administration (Figure 2). However, the ratio of intrafollicular E2 to retrieved follicles was higher in women with TC/CC genotypes than in women with the TT genotype (*p* < 0.02) (Figure 2). Our analysis of comorbidities between the two groups (TT and TC/CC genotypes) revealed no differences. There were no smokers in either group. Hypertension and diabetes were not found in any of these patients. The only comorbidity found was thyroid dysfunction. In all, 10% of the women with the TT genotype and 14% of the women with the TT/CC genotypes had thyroid dysfunction.

## Discussion

This work provides the first demonstration of an association between the *VDR TaqI* polymorphic C allele frequency and decreased follicle production by women exhibiting different causes of infertility. There were no significant differences in the genotype and allele frequencies of the *FokI* and *BsmI* polymorphisms between the COS and control groups. Instead, the higher frequencies observed in the group with polymorphic *TaqI* alleles in the COS group indicates that this *VDR* polymorphism is a potentially important SNP candidate that may be involved in female fertility.

There is some disagreement in the literature regarding the relationship between *VDR* polymorphisms and infertility disorders. Several studies have found that there is a negative association between the presence of *TaqI, FokI*, and *BsmI VDR* polymorphisms and the risk of developing reproductive disorders, such as polycystic ovarian syndrome and endometriosis (31–35). Conversely, other studies have found positive associations (36–38, 50) or no association at all (17) for these variables, suggesting that there is a need for further studies to clarify this important question.

We did not find an association between follicular 25(OH)D concentrations and any specific *TaqI* polymorphism allele (C or T), suggesting that these polymorphisms do not alter FF 25(OH)D concentrations. Importantly, while serum concentration of 25(OH)D were not evaluated in this study, recent findings reported by our (49) and other groups (13, 14, 43, 44, 51) have demonstrated that FF accurately reflects plasma 25(OH)D concentrations (14, 44) in both fertile and infertile patients.

The above results indicate a lack of a direct relationship between FF concentrations of 25(OH)D and infertility and suggest that the *TaqI* polymorphism does have a role in this context. In contrast, some studies have demonstrated an association between the *TaqI* C allele and decreased serum 25(OH)D concentrations in women with colorectal cancer (52), whereas another study performed in a healthy cohort in India (53) demonstrated that the *TaqI* C allele was directly associated with higher serum concentrations of 25(OH)D.

However, a study of polycystic ovarian syndrome in Caucasian women (32) found no association between *TaqI* polymorphic genotypes (TT, TC, CC) and 25(OH)D deficiency. This finding is in line with our results, given that we did not found any associations between *TaqI* polymorphism genotypes and intrafollicular 25(OH)D concentrations. Altogether, these data highlight the relevance of the *TaqI* polymorphism under different conditions and suggest the need for further studies investigating the relationship between *VDR* polymorphisms and 25(OH)D serum concentrations in different pathologies, including infertility disorders.

Our study has some limitations, including the relatively low number of included patients and the fact that we did not genotype all three *VDR* polymorphisms in all samples we evaluated. We also observed that there was a lack of HWE in the control population due to the exclusion criteria. This decreased the size of the study population and may have contributed to the observed imbalance in genotype and allele frequencies, resulting in a lack of HWE in the study populations. However, the COS population was under HWE and exhibited an association between the *TaqI* TC/CC polymorphic genotypes and the production of fewer ovarian follicles. These results suggest a possible role of the C allele in determining the number of pre-ovulatory follicles.

The above observation is supported by our data showing a higher ratio of retrieved follicles to intrafollicular E2 in women with TC/CC genotypes than in women carrying the TT genotype, i.e., women who have the TC/TT genotypes exhibited lower E2 availability in pre-ovulatory follicles. This finding demonstrates an important impact of the *TaqI* polymorphism on follicular development and hormone secretory function.

Recent studies have shown that 25(OH)D is present in FF (15, 19). While there is some controversy regarding the importance of FF 25(OH)D concentrations in positive IVF outcomes [for example, in patients with chemical pregnancies, embryonic implantation problems, chemical pregnancy (β-hCG level higher than 25 mIU/ml), in fertilization rates and in the numbers of embryos transferred and oocytes retrieved] (13, 14, 44), *VDR* mRNA and 1-α hydroxylase enzyme are expressed in the ovary (i.e., in ovarian cells and granulosa cell cultures) (12, 38), indicating that calcitriol activity affects local synthesis and autocrine and/or paracrine actions in the ovaries.

In conclusion, this study revealed an association between the presence of the C *VDR TaqI* polymorphism allele and infertility. This association is likely mediated by impaired calcitriol signaling, which may impact the number of follicles in women undergoing COS *via* mechanisms that are yet to be described.

## Ethics Statement

This study was approved by the local Ethics Committee of the Maternidade Escola of the Federal University of Rio de Janeiro, which was registered on the Brazilian platform of research under the number 02213812.4.0000.5275. All of the enrolled subjects (volunteers and patients) provided written informed consent before joining the study.

## Author Contributions

Conceptualization of the experiments. Formal analysis. Performed experiments. Writing review and editing.

## Conflict of Interest Statement

The authors declare that the research was conducted in the absence of any commercial or financial relationships that could be construed as a potential conflict of interest. The handling Editor and reviewer PP declared their involvement as co-editors in the Research Topic, and confirm the absence of any other collaboration.

## Supplementary Material

Figure S1 Agarose gel electrophoresis of DNA against a 100 bp DNA ladder (Promega) following PCR amplification and restriction fragment length polymorphism with the *TaqI* restriction enzyme. **(A)** Lane 1, uncut band with a length of 495 bp. Lanes 2, 3, 5, and 6 contain uncut 495 bp fragments, indicating that these samples have the TT (homozygous) genotype. Lane 4 contains two bands at 290 and 205 bp, indicating the CC (homozygous) genotype. **(B)** Lanes 1, 3, 4, 5, and 6 contain 495, 290, and 205 bp fragments, indicating the TC (heterozygous) genotype. Lane 2 contains an uncut band with a length of 495 bp. Lane 7 contains two bands at 290 and 205 bp, indicating the CC (homozygous) genotype.

Figure S2 Agarose gel electrophoresis of DNA against a 100-bp DNA ladder (Promega) following PCR amplification and restriction fragment length polymorphism with the *BseGI* restriction enzyme. **(A)** Lane 1 contains an uncut band at 265 bp. Lane 2 contains fragments at 265 bp, indicating the TT (homozygous) genotype. Lane 3 contains two bands at 196 and 69 bp, indicating the CC (homozygous) genotype. **(B)** Lane 1 contains an uncut band with a length of 265 bp. Lanes 2 and 3 contains fragments at 265 bp, indicating an uncut fragment and, therefore, the TT (homozygous) genotype.

Figure S3 DNA fragment sequences in affected and unaffected individuals. The BamH1 restriction site sequence is underlined, and the arrows indicate the polymorphic site. **(A)** DNA sequence electropherogram of the wild-type G/G genotype (arrow). **(B)** DNA sequence electropherogram of the homozygous A/A polymorphism (arrow). **(C)** DNA sequence electropherogram of the heterozygous G/A polymorphism (arrow).

## Abbreviations

BMI, body mass index; COS, controlled ovarian stimulation; FF, follicular fluid; E2, estrogen; P4, progesterone; FSH, follicle-stimulating hormone; GnRH, gonadotropin-releasing hormone; hCG, human chorionic gonadotropin; 1,25(OH)2D, 1,25-Hydroxycholecalciferol; 25(OH)D, 25-Hydroxyvitamin D; 25(OH)D3, 25-Hydroxycholecalciferol; 25(OH)D2, 25-Hydroxyergocalciferol; HWE, Hardy–Weinberg equilibrium; IVF, *in vitro* fertilization; ICSI, intracytoplasmic sperm injection*;* LH, luteinizing hormone; OR, odds ratio; RFLP, restriction fragment length polymorphism; SNP(s), single-nucleotide polymorphism (s); VDR, vitamin D receptor.
