# Supplementary figures and images for: Vitamin D Receptor TaqI Polymorphism Is Associated With Reduced Follicle Number in Women Utilizing Assisted Reproductive Technologies
Source: Front Endocrinol (Lausanne). 2018 May 28;9:252. doi: 10.3389/fendo.2018.00252 (PMC5985330; doi:10.3389/fendo.2018.00252)

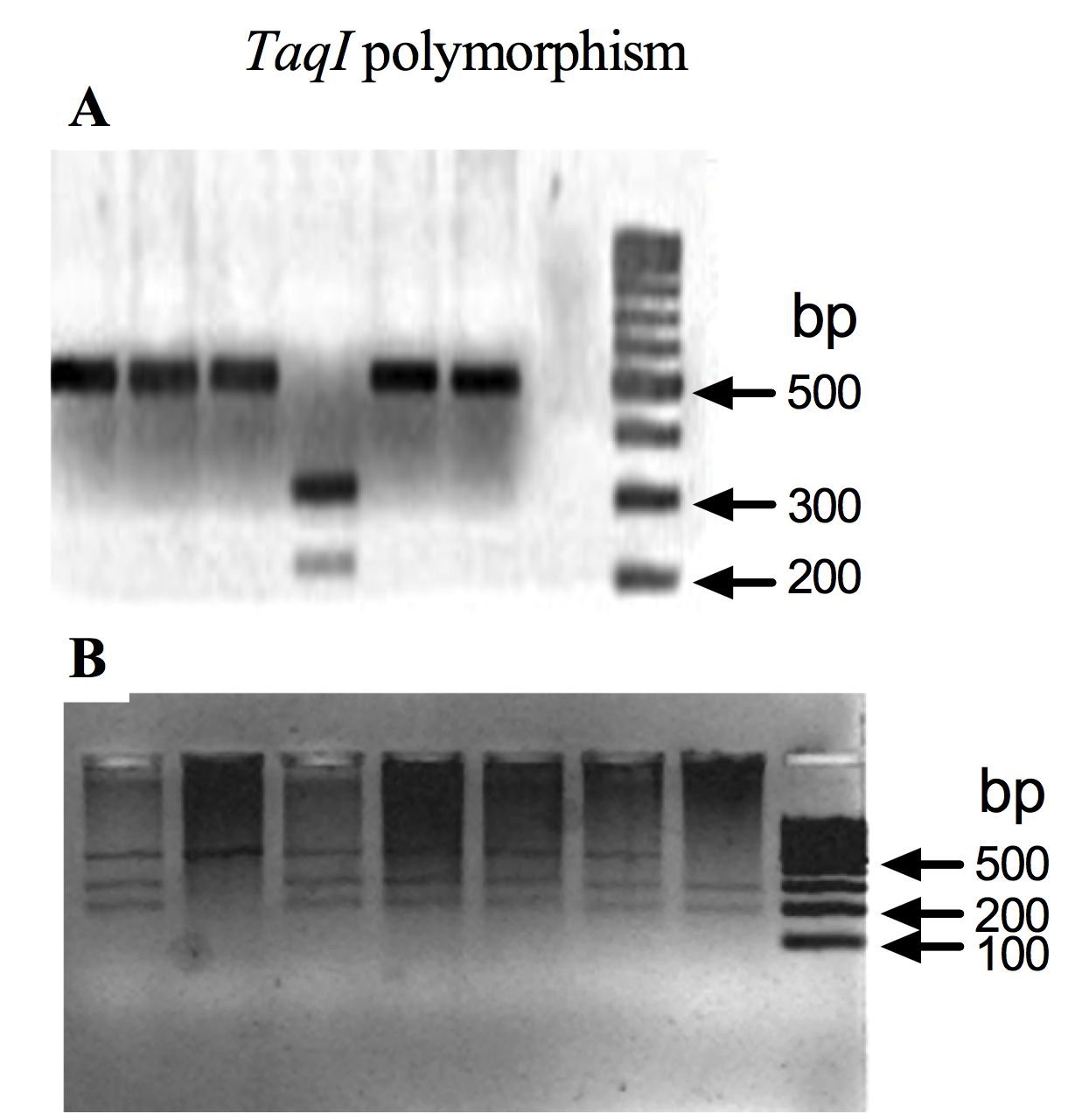

Supplement: Figure S1 — Agarose gel electrophoresis of DNA against a 100 bp DNA ladder (Promega) following PCR amplification and restriction fragment length polymorphism with the TaqI restriction enzyme. (A) Lane 1, uncut band with a length of 495 bp. Lanes 2, 3, 5, and 6 contain uncut 495 bp fragments, indicating that these samples have the TT (homozygous) genotype. Lane 4 contains two bands at 290 and 205 bp, indicating the CC (homozygous) genotype. (B) Lanes 1, 3, 4, 5, and 6 contain 495, 290, and 205 bp fragments, indicating the TC (heterozygous) genotype. Lane 2 contains an uncut band with a length of 495 bp. Lane 7 contains two bands at 290 and 205 bp, indicating the CC (homozygous) genotype. [file image_1.tiff]

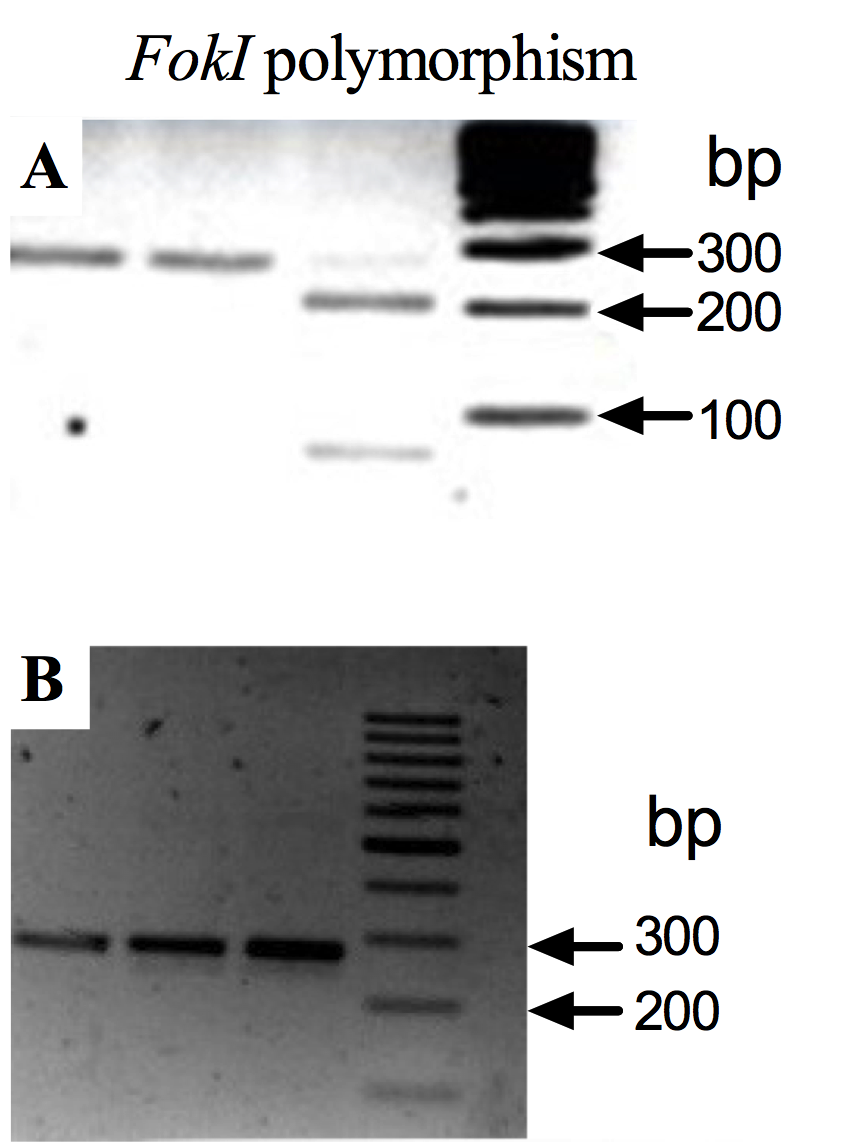

Supplement: Figure S2 — Agarose gel electrophoresis of DNA against a 100-bp DNA ladder (Promega) following PCR amplification and restriction fragment length polymorphism with the BseGI restriction enzyme. (A) Lane 1 contains an uncut band at 265 bp. Lane 2 contains fragments at 265 bp, indicating the TT (homozygous) genotype. Lane 3 contains two bands at 196 and 69 bp, indicating the CC (homozygous) genotype. (B) Lane 1 contains an uncut band with a length of 265 bp. Lanes 2 and 3 contains fragments at 265 bp, indicating an uncut fragment and, therefore, the TT (homozygous) genotype. [file image_2.tiff]

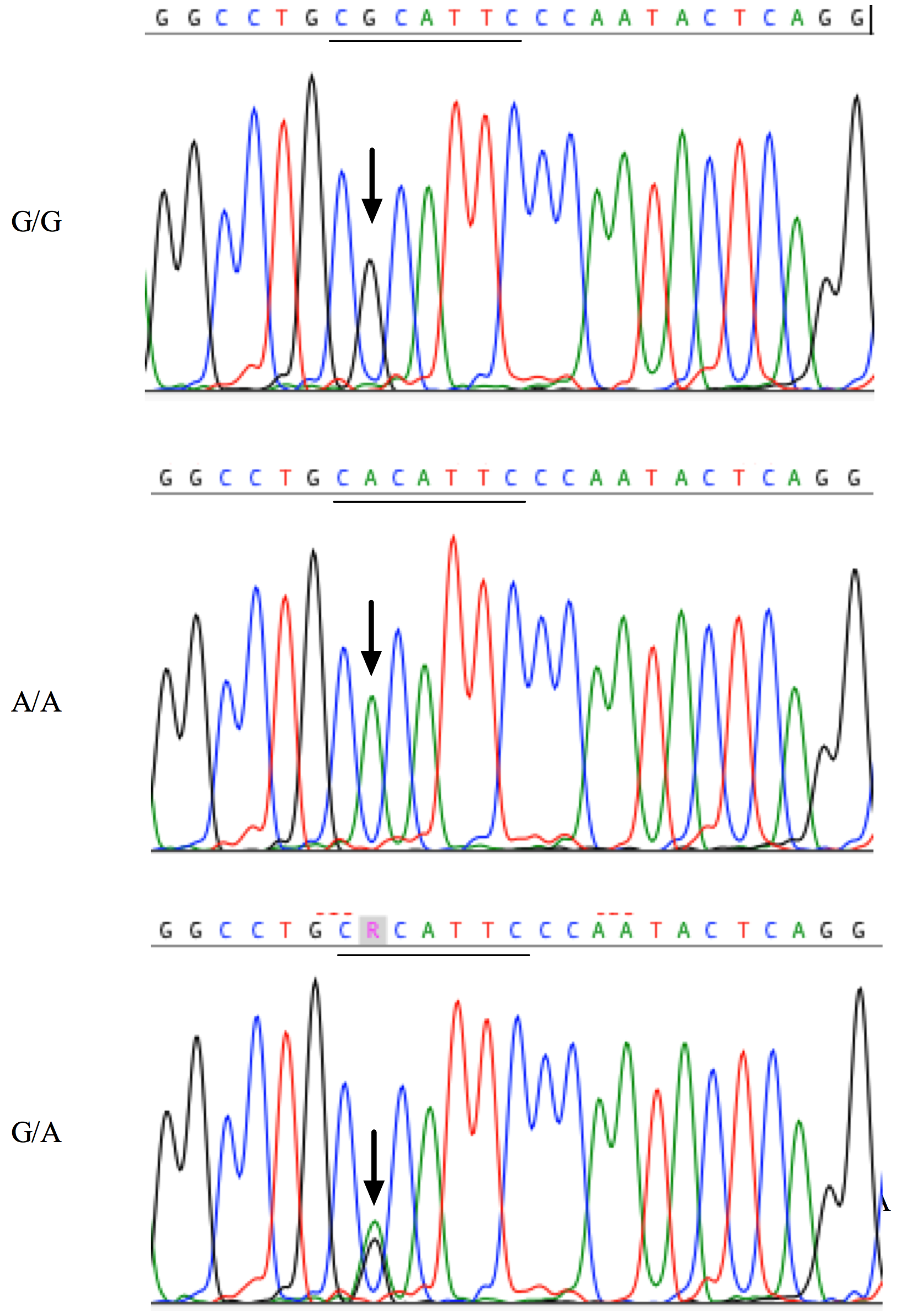

Supplement: Figure S3 — DNA fragment sequences in affected and unaffected individuals. The BamH1 restriction site sequence is underlined, and the arrows indicate the polymorphic site. (A) DNA sequence electropherogram of the wild-type G/G genotype (arrow). (B) DNA sequence electropherogram of the homozygous A/A polymorphism (arrow). (C) DNA sequence electropherogram of the heterozygous G/A polymorphism (arrow). [file image_3.tiff]
